# Prediction of kinase inhibitors binding modes with machine learning and reduced descriptor sets

**DOI:** 10.1038/s41598-020-80758-4

**Published:** 2021-01-12

**Authors:** Ibrahim Abdelbaky, Hilal Tayara, Kil To Chong

**Affiliations:** 1 Department of Electronics and Information Engineering, Jeonbuk National University, Jeonju, 54896 South Korea; 2 Agricultural Research Center, Giza, 12619 Egypt; 3 School of International Engineering and Science, Jeonbuk National University, Jeonju, 54896 South Korea; 4 Advanced Electronics and Information Research Center, Jeonbuk National University, Jeonju, 54896 South Korea

**Keywords:** Computational biology and bioinformatics, Drug discovery

## Abstract

Protein kinases are receiving wide research interest, from drug perspective, due to their important roles in human body. Available kinase-inhibitor data, including crystallized structures, revealed many details about the mechanism of inhibition and binding modes. The understanding and analysis of these binding modes are expected to support the discovery of kinase-targeting drugs. The huge amounts of data made it possible to utilize computational techniques, including machine learning, to help in the discovery of kinase-targeting drugs. Machine learning gave reasonable predictions when applied to differentiate between the binding modes of kinase inhibitors, promoting a wider application in that domain. In this study, we applied machine learning supported by feature selection techniques to classify kinase inhibitors according to their binding modes. We represented inhibitors as a large number of molecular descriptors, as features, and systematically reduced these features in a multi-step manner while trying to attain high classification accuracy. Our predictive models could satisfy both goals by achieving high accuracy while utilizing at most 5% of the modeling features. The models could differentiate between binding mode types with MCC values between 0.67 and 0.92, and balanced accuracy values between 0.78 and 0.97 for independent test sets.

## Introduction

Protein kinases are one of the largest enzyme families, representing about 2% of the human genome^1^. They are responsible for the phosphorylation process in which they catalyze the addition of phosphate groups to other proteins to make them functionally active. Phosphorylation relates kinases to many important biological processes in humans as it is important for cell division, signaling, and growth^2,3^.

Improper regulation of kinases had been clinically proven to be associated with different diseases including cancers, inflammatory and cardiovascular diseases^4,5^. This association assigned kinases a high significance from drug design perspective and ensured the need to study kinases when looking for a potential treatment for such diseases^6^. Given the important role of kinases, it is not surprising that the kinase family is currently involved in approximately 30% of the world’s drug discovery research efforts, making them one of the most important drug targets^5^.

X-ray crystal structures of kinase-inhibitor complexes have revealed details about the binding behaviors of these inhibitors. Inhibitors bind in different modes depending on specific binding locations and conformational aspects of the target kinase. Understanding these binding modes is important to analyze and improve the inhibition of kinases while looking for potential kinase-targeting drugs. The conformational aspects associated with the binding modes are determined by the states of two parts in the protein binding site. The first part is the Asp-Phe-Gly (DFG) motif, as part of the activation loop, and the second part is the $\alpha$C-helix. The DFG motif participates, through its -in/-out conformational states, in determining the active/inactive state of the kinase by closing/opening the activation loop and consequently the adenosine triphosphate (ATP) binding site^7^. The $\alpha$C-helix has also -in/-out conformational states. The $\alpha$C-helix-in state indicates an active kinase by helping the binding of the phosphate group in the ATP site. In its –out state, the $\alpha$C-helix moves out from its position resulting in an inactive kinase state. In summary, the conformational states of the DFG and $\alpha$C-helix lead to the kinase state becoming fully active (DFG-in, $\alpha$C-helix-in), or fully inactive (DFG-out, $\alpha$C-helix-out). Notably however, an intermediate conformational state (DFG-in, $\alpha$C-helix out), has been detected in some complexes^8^.

**Figure 1:**
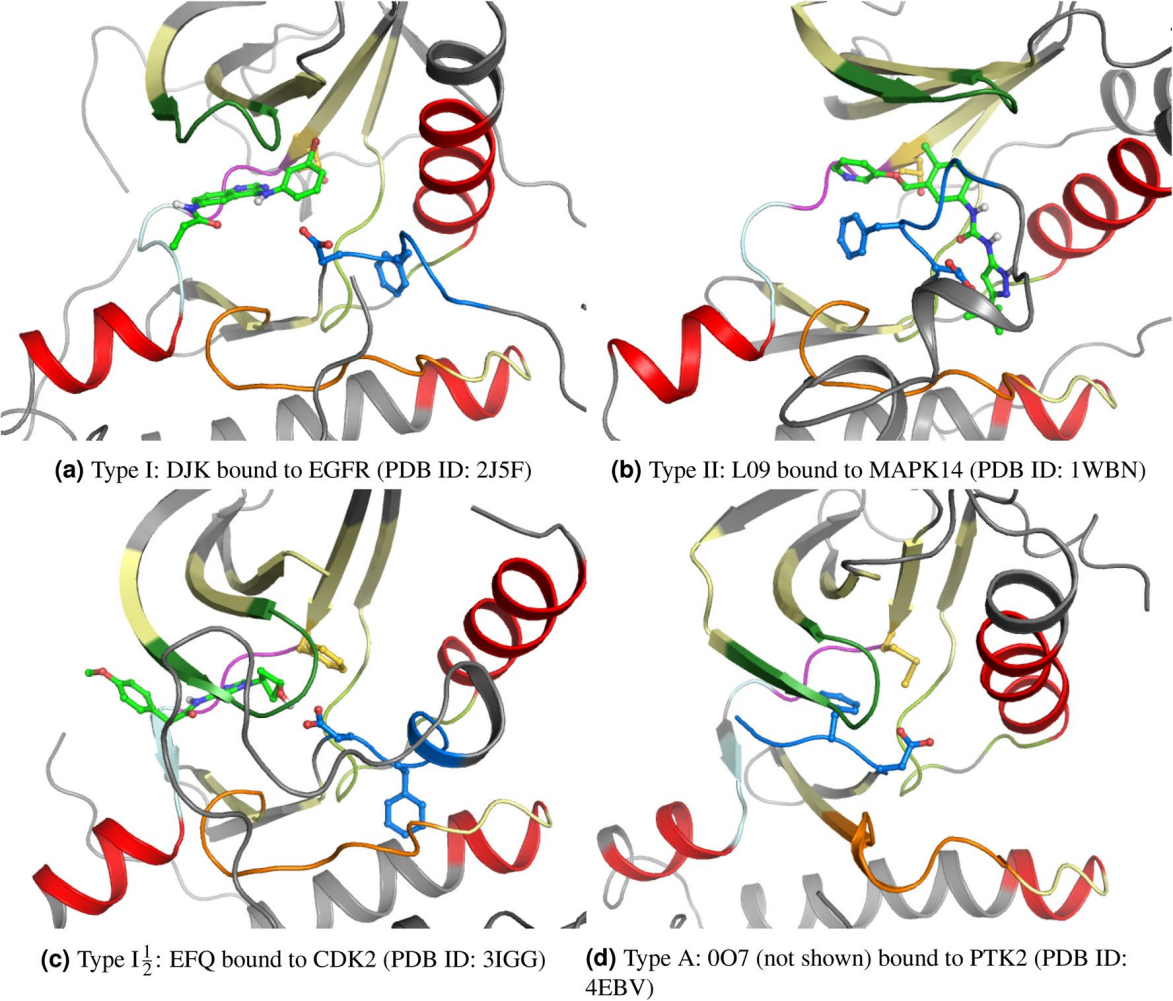
Snapshots, obtained from KLIFS^10–13^, for the binding site (with inhibitor) in each of the four binding modes (DFG: blue, $\alpha$C-helix: red (upper right), inhibitor: light green). (**a**) Type I: DJK bound to EGFR (PDB ID: 2J5F^14^), (**b**) Type II: L09 bound to MAPK14 (PDB ID: 1WBN^15^), (**c**) Type I$\frac{1}{2}$:EFQ bound to CDK2 (PDB ID: 3IGG^16^), and (**d**) Type A: 0O7 (not shown) bound to PTK2 (PDB ID: 4EBV^17^).

While there is a wide range of known kinase inhibitors, most of them inhibits kinases in the active form (conformational state: DFG-in, $\alpha$C-helix-in) and are known as type I binding mode inhibitors^8^. Type I inhibitors bind to the ATP binding site and elongate to nearby regions giving them some level of selectivity^6^. Type II inhibitors bind to a kinase in its inactive state (conformational state: DFG-out, $\alpha$C-helix-out). In this type, the inhibitors bind to the ATP binding site and extend to an adjacent allosteric back pocket that opens as a response to the (DFG-out, $\alpha$C-helix-out) conformation^6^. Another group of kinase inhibitors, type $I\frac{1}{2}$, binds in an intermediate state between the active and inactive forms (conformational state: DFG in, $\alpha$C-helix out)^9^. Finally, allosteric kinase inhibitors bind completely outside the ATP binding site in allosteric regions which could be adjacent (type III) or distant (type IV) with respect to the ATP site^8^. Figure 1 shows examples for the ATP binding site in each of the 4 binding modes. The snapshots are obtained from the structural kinase database, KLIFS^10–13^ (http://klifs.net/index.php), for structures with PDB IDs: 2J5F^14^, 1WBN^15^, 3IGG^16^, and 4EBV^17^.

In the course of inhibitor selectivity, type II inhibitors were anticipated to have high selectivity as they extend their binding outside of the ATP binding site. However, no experimental confirmation for this expectation has been made as there are selective and non-selective inhibitors of both type I and type II^18^. Some inhibitors of type I$\frac{1}{2}$ were found to be highly more selective for specific kinases, and allosteric inhibitors have the highest selectivity—more than all the ATP binding inhibitors^9,19^. Low selectivity makes it difficult to differentiate between inhibitors of different types. It is also difficult to structurally characterize each inhibitor case individually because of the large quantity of available data and the lack of complete structural characterization boundaries between binding modes, especially types I and II^20^. As a result, the task of determining inhibitor binding mode based on its structure is challenging.

Quantitative structure activity relationship (QSAR) modeling is a technique that assumes a relationship between the biological activity of compounds and their physicochemical and structural properties^21^. Compounds can be numerically represented based on their properties using molecular descriptors^22^ and thus they can undergo computational analysis predictive modelling.

The large amount of data about kinase inhibitors, 115000 inhibitors with a measured activity, made them a subject to data analysis and modelling in different studies^8^ There are many studies that used machine learning to target kinase related problems such as predicting kinase activity state^23^, and predicting bioactivity of compounds against mTOR kinase^24^, and others. However, few studies targeted the problem of predicting inhibitor binding modes. In^8^, they used different machine learning methods to construct 4 predictive models to distinguish between inhibitor binding modes of types I, II, I$\frac{1}{2}$, and allosteric. They based their predictions on data from X-ray crystal structures and inhibitor fingerprints that were calculated using compound Simplified Molecular Input Line Entry System (SMILES). They used 2 types of fingerprints: Extended Connectivity Fingerprints of diameter 4 (ECFP4) and Molecular ACCess System structural keys (MACCS). The SVM (Support Vector Machine) classifier was the best performing technique in their modelling. The models achieved high classification accuracy for 2 out of their 4 classification tasks, while the accuracy for the remaining 2 tasks was relatively low^8^. In^25^, they worked to improve differentiation ability between non-allosteric inhibitors of types I, II, and I$\frac{1}{2}$. They built predictive machine learning models with an active learning strategy. They used different representations, including compound (ECFP4) and protein-ligand Interactions Fingerprints (IFPs), individually and in combination. The accuracy was relatively improved over their previous work^25^.

Motivated by the large quantity of available data, there is an increased emphasis on the role of computational techniques for understanding the behavior of kinase inhibitors. In this study, we worked to achieve better predictions for kinase inhibitor binding modes by applying a variety of machine learning and feature selection techniques. Instead of fingerprints, we calculated different types of molecular descriptors and used them to represent the known kinase inhibitors. The sets of descriptors were reduced using different feature reduction techniques and then employed to build machine learning classifiers. The developed predictive models could differentiate between kinase inhibitors belonging to different binding modes with a higher accuracy compared to the only 2 previous studies in this domain.

## Results and discussion

**Figure 2:**
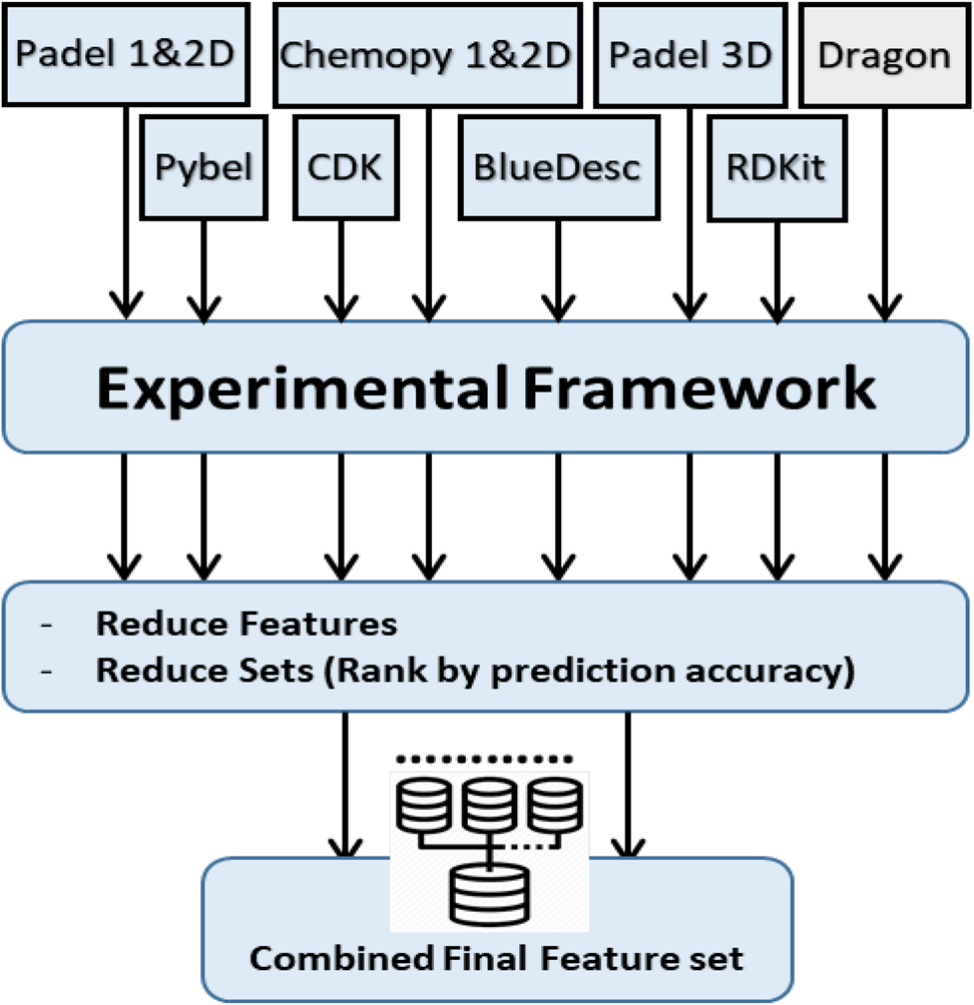
Testing and combining individual descriptor sets.

In this section we present the results obtained for the different classification tasks. In addition to the modeling results, we mention the feature sets used for each classification task and the number of total features that gave the best results. For each classification task, we first performed the workflow on each individual descriptor set, i.e. 8 times for each task. We then combined the best performing descriptors that produced good predictions for each task. The final prediction model for each task was based on the combinations of 4 or 5 descriptor sets based on individual set results, Fig. 2.

### Individual set experiments

For each classification task: after applying our experimental methodology on each of the 8 descriptor sets individually, we obtained a selected list of features from each set. Some of these lists showed high potential for specific classification tasks, while others showed low, or no, prediction potential. We mention in Table 1 the results and the descriptor set of the highest predictive lists for each task. Full results for all descriptor sets and classification tasks are shown in the supplementary material (Table S1). Evaluation and comparisons were based on the Mathews Correlation Coefficient (MCC).

**Table 1:** The best predictive descriptor set for each classification task.

CT	Desc. Set*	Sel. Desc.*	MCC Valid.*	MCC Ind.*
$CT_{I-II}$	Dragon	185	0.87	0.74
$CT_{I-I\frac{1}{2}}$	Padel 3D	39	0.86	0.87
$CT_{II-I\frac{1}{2}}$	Chem 1&2D	68	0.78	0.77
$CT_{A-(I+II+I\frac{1}{2})}$	Dragon	197	0.54	0.49

Desc. Set*: Descriptor Set; *: No. of selected descriptors; MCC valid.*: MCC for validation set; MCC Ind.*: MCC for independent set.

### Combined sets experiments

We tried different combinations of the selected lists of descriptors to determine the most efficient overall feature set, for each task. We found that the best performing combination, for each classification task (CT), did not exceed 5 types of descriptors. The best combination of selected descriptors for the different classification tasks were as follows:

- $CT_{I-II}$: The combination of the best 5 selected lists of descriptors included: Padel 1&2D, Padel 3D, Chemopy 1&2D, RDKit, and Dragon. This combination led to an improved prediction MCC over the individual Dragon set by 0.05 for the validation test with 110 final combined descriptors.
- $CT_{I-I\frac{1}{2}}$: The combination of the best 4 selected lists of descriptors included: Padel 1&2D, Padel 3D, Chemopy 1&2D, and Dragon. This combination led to an improved prediction MCC over the individual Padel 3D set by 0.03 and 0.01 for the validation and independent tests respectively with 183 final combined descriptors.
- $CT_{II-I\frac{1}{2}}$: The combination of the best 4 selected lists of descriptors included: Padel 1&2D, Padel 3D, Chemopy 1&2D, and Dragon. This combination led to an improved prediction MCC over the individual Chem 1&2D set by 0.2 and 0.15 for the validation and independent tests respectively, with 50 final combined descriptors.
- $CT_{A-(I+II+I\frac{1}{2})}$: The combination of the best 5 selected lists of descriptors included: Padel 1&2D, Padel 3D, Chemopy 1&2D, RDKit, and Dragon. This combination led to an improved prediction MCC over the individual Dragon set by 0.24 and 0.19 for the validation and independent tests respectively, with 215 final combined descriptors.

Table 2 shows the final results for the validation and independent test sets for different classification tasks. The values represent the averages over the 10 validation experiments. We used three metrics for evaluating the models: F1 measure, Mathews Correlation Coefficient (MCC), and Balanced Accuracy (BA).

From the results in Table 2, we can see that the predictive models in the classification tasks for non-allosteric inhibitors always showed high accuracy. The F1 measure was between 0.94 and 0.98 showing a high ability in identifying the positive classes which were types I and II, in non-allosteric tasks. For $\text {CT}_{II-I\frac{1}{2}}$, the F1 value was 0.65, considering the few number of samples in the positive class (type A), this value denotes a promising prediction, although less than other CTs. BA values were always over 75%, with the highest value achieved by the model for $\text {CT}_{II-I\frac{1}{2}}$: 97%. Models for all tasks performed well and without being biased to the majority class despite the imbalance in training data. MCC values were the most important as it has high confidence in imbalanced models validation. The values for MCC were between 0.67 and 0.92 showing a high predictive ability for the models in all tasks for both classes. The average confusion matrix shows few number of misclassified items. The highest rate of misclassification had the relative ratio of 5.3% in $\text {CT}_{I-II}$. CTs that contained the data samples of type I$\frac{1}{2}$ inhibitors achieved the highest accuracies in all metrics, suggesting that type I$\frac{1}{2}$ as being easier to identify. The results for the (80–20%) splits are shown in the supplementary material (Table S2).

We used mainly Support Vector Machines (SVM) for classification. In addition, we tested the performance of different machine learning techniques such as Random Forests (RF), Gradient Boosting (GB), and Linear Regression (LR) models based on the final selected features for different classification tasks. LR results were comparable to SVM and confirming the efficiency of the selected features. In general for all classification tasks, SVM was found to be the best performing classifier as shown in the full results, supplementary material (Table S3).

**Table 2:** Evaluation results for different classification tasks on validation and independent sets.

CT	Eval. metric	Validation	Independent
$\text {CT}_{I-II}$	F1	0.99 (± 0.01)	0.97 (± 0.003)
	BA	0.94 (± 0.01)	0.85 (± 0.01)
	MCC	0.92 (± 0.02)	0.74 (± 0.02)
	Confusion matrix (%)	$$\begin{bmatrix} 10.5 &{} 1.3 \\ 0.4 &{} 87.8 \\ \end{bmatrix} \quad $$	$$\begin{bmatrix} 8.6 &{} 3.2 \\ 2.1 &{} 86.1 \\ \end{bmatrix} \quad $$
$\text {CT}_{I-I\frac{1}{2}}$	F1	0.99 (± 0.00)	0.98 (± 0.00)
	BA	0.96 (± 0.01)	0.93 (± 0.01)
	MCC	0.94 (± 0.01)	0.88 (± 0.01)
	Confusion matrix (%)	$$\begin{bmatrix} 19.9 &{} 1.8 \\ 0.4 &{} 77.9 \\ \end{bmatrix} \quad $$	$$\begin{bmatrix} 18.9 &{} 2.9 \\ 0.9 &{} 77.3 \\ \end{bmatrix} \quad $$
$\text {CT}_{II-I\frac{1}{2}}$	F1	0.98 (± 0.01)	0.94 (± 0.01)
	BA	0.99 (± 0.01)	0.97 (± 0.01)
	MCC	0.98 (± 0.01)	0.92 (± 0.02)
	Confusion matrix (%)	$$\begin{bmatrix} 66.9 &{} 0.6 \\ 0.4 &{} 32.1 \\ \end{bmatrix} \quad $$	$$\begin{bmatrix} 64.4 &{} 3.1 \\ 0.8 &{} 31.7 \\ \end{bmatrix} \quad $$
$\text {CT}_{A-(I+II+I\frac{1}{2})}$	F1	0.77 (± 0.07)	0.65 (± 0.12)
	BA	0.82 (± 0.05)	0.78 (± 0.08)
	MCC	0.78 (± 0.07)	0.67 (± 0.11)
	Confusion matrix (%)	$$\begin{bmatrix}97.6 &{} 0.1 \\ 0.8 &{} 1.5 \\ \end{bmatrix} \quad $$	$$\begin{bmatrix} 97.5 &{} 0.3 \\ 1.0 &{} 1.2 \\ \end{bmatrix}$$

### Final features

The final number of selected features for each task is shown in Table 3. The table shows also the portions of final features that belong to different descriptor sets, along with their relative percentages. For further examination of the selected features, we extracted their sub-types. The number of final features in each sub-type is shown in Table 4. The counts shown are the grouped counts over all classification task results. Features selected from Dragon descriptors mostly lie in the GETAWAY and Functional group count descriptors. From the Padel set, most final descriptors are in the E-state, RDF (Radial Distribution Functions), and Topological descriptors. For Chemopy 1&2D, MOE-type (Molecular Operating Environment) and Autocorrelation descriptors represented the largest portion. Finally, most of the selected RDKit descriptors belong to the Constitutional descriptors. A list of the final selected descriptors for each classification task is available in the supplementary material (Tables S4:S7). We examined the frequently selected functional group descriptors and listed them in the supplementary material (Table S8). We show some examples of good and poor predictions by our models for each type in the supplementary material (Table S9). In (Table S9), structures were retrieved from PubChem database^26^ (https://pubchem.ncbi.nlm.nih.gov/) for 8 example compounds with PubChem identifiers (CID): 71737839, 45139233, 66563698, 11288934, 445840, 122235220, 129900107, and 46897873.

**Table 3:** Number of selected descriptors from each set in the classification tasks.

CT	Descriptor set	Final no.	% of final	Total feat.
$\text {CT}_{I-II}$	Padel 1&2D	31	28	110
	Padel 3D	12	11	
	Chemopy 1&2D	14	13	
	RDKit	7	6	
	Dragon	46	42	
$\text {CT}_{I-I\frac{1}{2}}$	Padel 1&2D	51	28	183
	Padel 3D	20	11	
	Chemopy 1&2D	28	15	
	Dragon	84	46	
$\text {CT}_{II-I\frac{1}{2}}$	Padel 1&2D	9	18	50
	Padel 3D	11	22	
	Chemopy 1&2D	18	36	
	Dragon	12	24	
$\text {CT}_{A-(I+II+I\frac{1}{2})}$	Padel 1&2D	65	30	215
	Padel 3D	18	8	
	Chemopy 1&2D	28	13	
	RDKit	18	8	
	Dragon	86	40

**Table 4:** Number of final descriptors grouped over all tasks by sub-type.

Descriptor set	Descriptor sub-type	Subset total	Set total
Dragon	GETAWAY descriptors	38	228
	Functional group counts	36	
	3D-MoRSE descriptors	32	
	RDF descriptors	30	
	Atom-centred fragments	29	
	WHIM descriptors	13	
	Topological	13	
	Others(9)	37	
Padel (All) (1D+2D+3D)	E-state descriptors	41	224
	RDF descriptors	36	
	Topological descriptors	35	
	Autocorrelation descriptors	34	
	3D Autocorrelation descriptors	23	
	Burden descriptors	19	
	Others(10)	36	
Chemopy 1&2D	MOE-type Descriptors	22	103
	Autocorrelation Descriptors	22	
	E-state Descriptors	14	
	Charge Descriptors	10	
	Others(6)	13	
RDKit	Constitutional descriptors	18	25
	Others(2)	7	

### Results visualization

**Figure 3:**
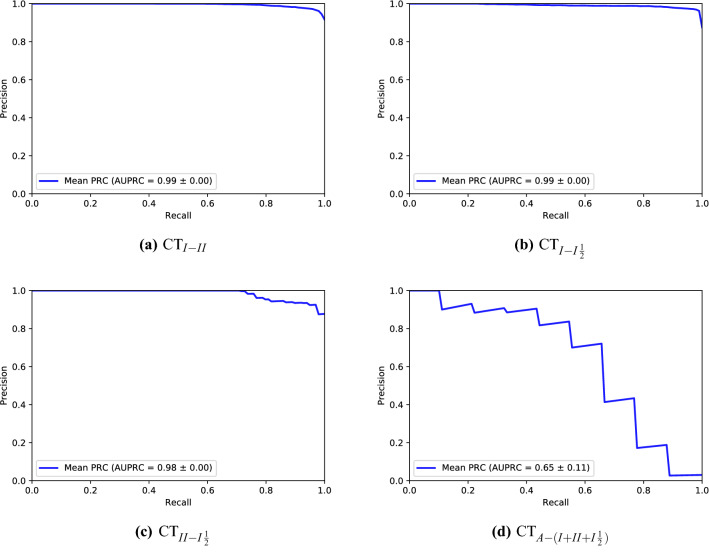
Average precision recall curves for the independent test sets in classification tasks. (**a**) $\text {CT}_{I-II}$, (**b**) $\text {CT}_{I-I\frac{1}{2}}$, (**c**) $\text {CT}_{II-I\frac{1}{2}}$, and (**d**) $\text {CT}_{A-(I+II+I\frac{1}{2})}$.

**Figure 4:**
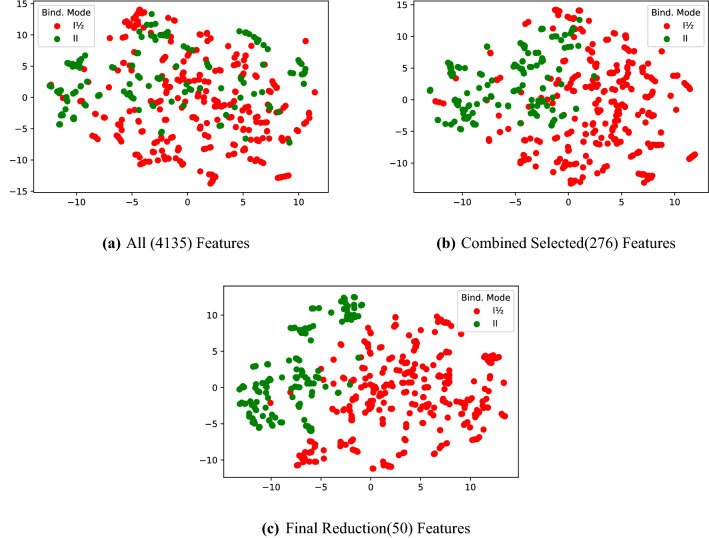
t-SNE plots for the features in classification task $\text {CT}_{II-I\frac{1}{2}}$. (**a**) All: 4135 features, (**b**) combined selected: 276 features, (**c**) final reduction: 50 features.

Figure 3 shows the average precision recall curves for the 10 validation experiments (independent sets) for each classification task. The curve exhibits high prediction ability for the first three models, while showing less prediction ability in the fourth $CT_{A-(I+II+I\frac{1}{2})}$ model. This low prediction ability is likely the result of the high data imbalance and the low number of available positive samples. Receiver Operating Characteristic (ROC) curves for the final models were plotted, and the areas under the ROC curves were calculated, in the supplementary material (Figure S1). The selected features demonstrated a good ability to differentiate between positive and negative classes. The t-SNE plot in Fig. 4 shows, as an example, how the discriminative ability for the predictive features in classification task $CT_{I-I\frac{1}{2}}$ was improved after final feature reduction.

### Comparison to previous work

We compared our results to the work of Miljkovic et al.^8^, as it was the only study that targeted the same domain for classifying all binding mode types. Table 6 shows a compassion between our newly proposed models and their models. Our models could achieve more accurate predictions in different classification tasks. The improvement in F1 score ranged between 17 and 40%, while BA was improved by 5–19%, and finally, the MCC was improved by 4–41%. The classification of allosteric and non-allosteric inhibitors, $CT_{A-(I+II+I\frac{1}{2})}$ , was the most challenging because of the scarcity of data for allosteric inhibitors. The feature selection methodology could lead to improved prediction for this task although only few number of positive samples were available. The improvement for this task was 29% in F1, 15% in BA, and 19% in MCC. In addition, a remarkable improvement could be noticed in the classification task $CT_{I-I\frac{1}{2}}$ as F1 was improved by 40%, BA was improved by 19%, and MCC was improved by 41%.

### Case studies

In order to evaluate the applicability of our models, we obtained and tested five new kinase inhibitors as case studies: L1H, JWY, 9NQ, 1LT, and 8OR. These inhibitors were not present in the original dataset that we used for model building. We calculated their descriptors and tested our models using the descriptor values required for each corresponding classification task, then we evaluated the predictions against the corresponding entries in KLIFS database. The $CT_{A-(I+II+I\frac{1}{2})}$ model could predict all the compounds correctly as non-allosteric. Classification task models $CT_{I-I\frac{1}{2}}$ and $CT_{II-I\frac{1}{2}}$ could correctly identify types I and II respectively. On the other hand, the model for $CT_{I-II}$ could correctly identify 9NQ, 1LT, 8OR as type I, and JWY as type II, while it incorrectly identified L1H as type II inhibitor. The prediction results for the five case study inhibitors are shown in Table 5.

**Table 5:** Results for predicting case study inhibitors.

Inhibitor	Real type	Model prediction			
		$\text {CT}_{I-II}$	$\text {CT}_{I-I\frac{1}{2}}$	$\text {CT}_{II-I\frac{1}{2}}$	$\text {CT}_{A-(I+II+I\frac{1}{2})}$
L1H	I	II	I	NA	Other
JWY	II	II	NA	II	Other
9NQ	I	I	I	NA	Other
1LT	I	I	I	NA	Other
8OR	I	I	I	NA	Other

L1H was incorrectly predicted as type II while it was characterized as type I inhibitor in its complex with EFGR kinase in KLIFS. By an initial inspection, we found that the binding pocket sequence in the structure containing L1H (PDB ID: 6S9B^27^) differs in 2 residues, T790M (Gatekeeper) and L858R, from other EFGR-inhibitor structures that are characterized as type I (2ITN^28^). These differences were found in some other EFGR-inhibitor structures that were characterized as type II (5UGC^29^). While we cannot conclude this as the only reason for the incorrect prediction, we strongly suggest that further inspection that takes protein sequence and structure information into consideration could result in improved predictive models for the binding modes.

**Table 6:** Proposed models results compared to the results in Miljkovic et al.^8^ for independent test sets in the 4 classification tasks.

CT	Metric	Proposed	Miljkovic et al.
$\text {CT}_{I-II}$	F1	0.97 (± 0.00)	0.71 (± 0.03)
	BA	0.85 (± 0.01)	0.78 (± 0.02)
	MCC	0.74 (± 0.02)	0.70 (± 0.04)
$\text {CT}_{I-I\frac{1}{2}}$	F1	0.98 (± 0.00)	0.58 (± 0.04)
	BA	0.93 (± 0.01)	0.74 (± 0.02)
	MCC	0.88 (± 0.01)	0.47 (± 0.05)
$\text {CT}_{II-I\frac{1}{2}}$	F1	0.94 (± 0.01)	0.77 (± 0.03)
	BA	0.97 (± 0.01)	0.82 (± 0.02)
	MCC	0.92 (± 0.02)	0.69 (± 0.03)
$\text {CT}_{A-(I+II+I\frac{1}{2})}$	F1	0.65 (± 0.12)	0.36 (± 0.18)
	BA	0.78 (± 0.08)	0.63 (± 0.07)
	MCC	0.67 (± 0.11)	0.48 (± 0.09)

## Materials and methods

In this section we will describe the data we used in our study. In addition, we will present the details of our workflow to produce the binding mode predictive models.

### Dataset and descriptors

#### Inhibitors dataset

In our study we used a dataset of compound SMILES that represent previously identified kinase inhibitors categorized by their known binding mode. The dataset was obtained from the previous study in^8^, and was originally extracted from the KLIFS database^10–13^, a repository that contains a wealth of characteristics for kinase-inhibitor complexes in the Protein Data Bank (PDB)^30^. The inhibitors were primarily retrieved based on binding site preference: allosteric or non-allosteric. The non-allosteric inhibitors were then classified into 3 categories based on their binding to the ATP or nearby sites. The result is a total of 4 inhibitor groups divided according to their binding mode: Type I, type II, type I$\frac{1}{2}$ , and type A, replicating the division in^8^. The records in the dataset is composed of an identifier, SMILES code, and binding type code. The majority of the inhibitors belonged to the first class, type I, with 1420 (69.2%) entries. The least number of items was in the allosteric class (A), with only 47 (2.3%) entries. While Types II and $I\frac{1}{2}$ had 190 (9.3%) and 394 (19.2%) inhibitors, respectively. The total number of inhibitors was 2051. SMILES in each group were used to calculate molecular descriptors.

#### Molecular descriptors

We used molecular descriptors to represent the features for inhibitor compounds. We utilized a comprehensive set of descriptors calculated through a variety of methods, including physiochemical properties and structural 1D, 2D and 3D descriptors. Eight sets of descriptors were calculated using two online webservers. ChemDes^31^ was used to calculate 7 sets of descriptors: Padel 1&2D, Padel 3D, Chemopy 1&2D, RDKit, Pybel, CDK, and BlueDesc, while e-Dragon^32,33^ was used to calculate an additional set. The total number of calculated descriptors was 4933. The number of descriptors in each set is shown in Table 7. Some descriptors were repeated across different sets and we handled this redundancy by dropping highly correlated features as we will show later.

**Table 7:** Descriptor sets.

Descriptor set	No. of descriptors
Padel 1&2D	1544
Padel 3D	431
Chemopy 1&2D	633
RDKit	196
Pybel	14
CDK	275
BlueDesc	174
Dragon	1666
Total	4933

### Methods

In order to classify inhibitors according to their binding mode, we constructed 4 classification tasks representing 4 pairs of classes. For simplicity, we will label a classification task between classes ***i*** and ***j*** as ***CTi-j***. The 4 classification tasks are:

1. ($CT_{I-II}$): To distinguish between types I and II inhibitors.
2. ( $CT_{I-I\frac{1}{2}}$): To distinguish between types I and I$\frac{1}{2}$ inhibitors.
3. ($CT_{II-I\frac{1}{2}}$): To Differentiate between types II and I$\frac{1}{2}$ inhibitors.
4. ($CT_{A-Others}$)): To identify allosteric inhibitors, type A, from non-allosteric, types I; II; and I$\frac{1}{2}$ inhibitors — the most challenging dataset with high imbalance.

The inhibitor classes in the four classification tasks with their details are shown in Table 8.

**Table 8:** Details of the classification tasks.

Classification task	Positive class		Negative class	
	Type	Count	Type	Count
$CT_{I-II}$	Type I	1420	Type II	190
$CT_{I-I\frac{1}{2}}$	Type I	1420	Type $I\frac{1}{2}$	394
$CT_{II-I\frac{1}{2}}$	Type II	190	Type $I\frac{1}{2}$	394
$CT_{A-Others}$	Type A	47	(Type I + Type II + Type $I\frac{1}{2}$)	2004

**Figure 5:**
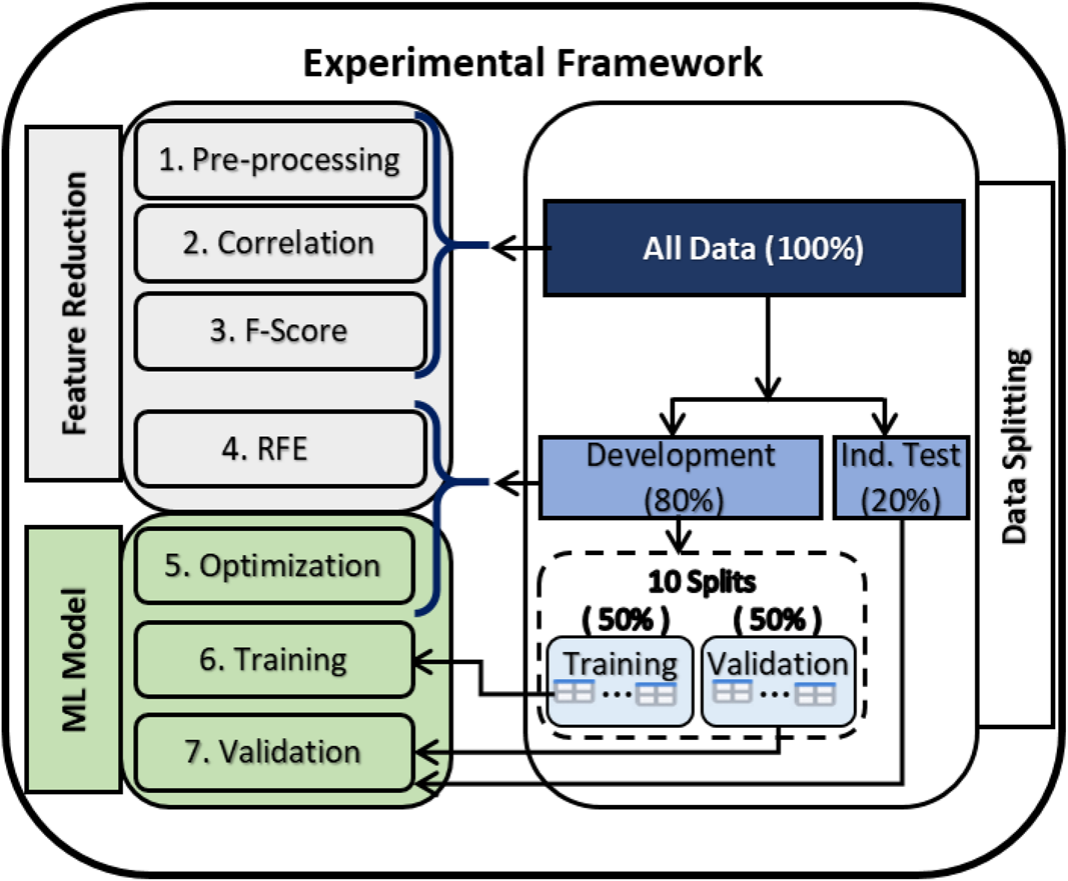
Data reduction and model building work flow in the proposed experimental framework.

Data Splits: For each classification task, the data were split in correspondence to different processing and reduction steps as shown in Fig. 5. All data passed through the cleaning, correlation, and f-score reduction steps. Then, we first split the data to 80% for development, and 20% for independent testing. The 80% was used for the Recursive Feature Elimination (RFE) feature selection, model development, and with further splitting for the validation. In the second splitting for validation, we adopted the splitting strategy in^8^ to allow comparison of the final results. We did split the development set 10 different times to training and validation sets using 50–50% splits and controlled by using different random states. In addition, we also performed further validation in the same manner but using splits of ratios 80–20%.

We followed the data and work flows shown in Fig. 5 for each of the 4 presented classification tasks on 2 levels: for individual descriptor set level, and for combined selected descriptors level.

**Figure 6:**
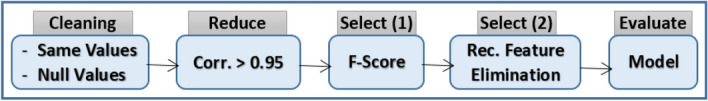
Feature processing and reduction flow.

A simple flow of steps representing data prepossessing, reduction, and modelling, is shown in Fig. 6.

#### Data pre-processing

In this step, the dataset for each pair in classification tasks was cleaned by dropping features that had:

- Zero variant values.
- Any empty values.

#### Correlation feature reduction

Descriptors with mutual correlation over 0.95 were reduced by keeping only one of them, and dropping the other, in order to prevent redundancy and to eliminate feature repetition. The correlation score was determined using Pearson correlation^34^.

#### F-score feature selection

F-score feature importance was calculated for each feature to decide its probable discriminative ability. F-score is a simple feature ranking technique that measures the distinction between two sets of real values (feature/target), but ignoring mutual information among different features. It assigns each feature in the dataset a value, while a higher f-score value denotes a more important feature^35,36^. In our work, we listed the features in a descending order based on their assigned F-score value. Then we created different feature partitions. Each partition contains a percentage of the whole features. For example: partition1: 100%, partition 2: top 95%, partition 3: top 90%, and so on. After this division, we continue our experimental framework using one partition at a time and then comparing the outputs to identify the best predictive set of features. There was no clear correlation between the number of features in each partition and the prediction accuracy. Each data set had its independent threshold that was identified through different trials. In general for all classification tasks, using F-score selection resulted in faster modeling and better predictions over the cases of being omitted (using 100% features).

#### Recursive feature elimination selection

The third feature reduction step was the Recursive Feature Elimination (RFE) method proposed in^37^ for ranking and selecting features by iterative training of an SVM model. The model assigns a ranking for features and recursively drops low-ranked features and keeps important ones^38^. We applied this step using scikit-learn implementation of RFE, RFECV, combined with SVM linear classifier. The RFECV assigns important features the value of 1, which we keep for model training, and non-important features the value of 0, which we drop from our modelling features. Based on the final feature set determined by the RFE method, we reduced the number of features in our development and independent test sets, keeping only the RFE selected feature list. The resulting dataset from this step was the one used for model training and validation.

#### Model development and optimization

We built binary classification models for predictions. In binary classification, the model (classifier) works to assign one of two class labels to the input pattern. One class is marked as positive, while the other is marked as negative^38^. An SVM^39^ binary classifier model was originally selected guided by previous results in the domain. For more confidence, we also trained binary classifiers of different types to test the performance of the final selected features. Random forest (RF), gradient boosting (GB), and linear regression (LR) models were tested. The training set was in the form of patterns of descriptor values with a known binding mode label. Our SVM models were built using the minimized datasets with 50–50% training-validation splits. The model parameters were optimized using an initial split, and the resulting best model was then tested and validated using the 10-splits strategy.

Model Optimization: SVM parameters can be tuned in order to reduce overfitting. The kernel parameter determines the type of the hyperplane, linear or non-linear. The gamma parameter is used with non-linear hyperplanes and higher values indicate near to exact fit to training data but this can lead to overfitting. The C parameter represents the error penalty, and it controls the balance between correct classification and a smoother decision boundary^40^. The initial SVM classifier model was optimized using the grid search optimization method in scikit-learn. We provided different values for the parameters, including different kernel types. The resulting optimized models had a slight difference in the best parameter values. The selected kernel was always the ‘rbf’ kernel. The optimized values for C were 100 for $CT_{I-II}$ and $CT_{II-I\frac{1}{2}}$, 150 for $CT_{I-I\frac{1}{2}}$, and 200 for $CT_{A-(I+II+I\frac{1}{2})}$. The gamma selected value was always ‘auto’, while the degree value was not considered in the selection as it is related only to the ‘poly’ kernel.

#### Model training

We did fit the optimized SVC model 10 different times using a different training set from the 10-splits described earlier. After each fit, the model accuracy is evaluated using the corresponding validation set, in addition to the independent test set.

#### Model validation

Model validation was performed based on the average value of each evaluation metric calculated over the 10 experiments for both validation and independent test sets.

Evaluation Metrics: In order to evaluate the model’s performance in each classification task, we used a variety of evaluation metrics. Taking into consideration the imbalance in the datasets for the classification tasks, we used balanced accuracy (BA) and Mathews Correlation Coefficient (MCC) in addition to the traditional F1 measure. The F1 measure, Eq. (1), is highly dependent on the positive class and it completely ignores the true negative rate. This could give misleading estimations when the positive class is accurately predicted but the negative class is poorly predicted. To handle this problem in our evaluation, we used also balanced accuracy and MCC as they could account for imbalanced data patterns.

1 $$\begin{aligned} F1 = \frac{2*TP}{2*TP+FP+FN} \end{aligned}$$

Balanced Accuracy (BA) overcomes regular accuracy problems as it accounts for imbalanced classes^24,41^. It is calculated based on the confusion matrix as shown in Eq. (2).

2 $$\begin{aligned} BA = \frac{1}{2}\left( \frac{TP}{TP+FN}+ \frac{TN}{TN+FP}\right) \end{aligned}$$

Mathew’s correlation coefficient (MCC) is another widely used metric that has a high level of confidence and is considered the most important indicator when a training dataset is imbalanced^24^. MCC can be calculated from the confusion matrix as in Eq. (3). The range of the MCC values is between − 1, for complete misclassification, and 1, for complete classification^42,43^

3 $$\begin{aligned} MCC = \frac{(TP*TN) - (FP*FN)}{\sqrt{(TP+FP)(TP+FN)(TN+FP)(TN+FN)}} \end{aligned}$$

In Eqs. (1) – (3), TP is the true positive, FP is the false positive, TN is the true negative, and FN is the false negative.

## Conclusion

In this paper, we computationally investigated the rarely investigated domain of kinase inhibitor binding modes. We aimed to discriminate between four modes: 3 allosteric inhibitor modes (I, II, I$\frac{1}{2}$) and one nonallosteric mode (A). We combined feature selection and machine learning methods to achieve efficient binding mode predictions using reduced feature sets. We used a wide range of calculated molecular descriptors as features, and these were collected from 8 descriptors sets, totaling 4933 descriptors. Features were initially reduced by cleaning and dropping high correlations. Further reduction was done using the F-score and RFE techniques to find the most important features for efficient modeling. Different machine learning classifiers were optimized and evaluated on the dataset with the reduced set of features. The final best performing models were SVM models as they could achieve accurate predictions with less than 5% of the original number of features. Previous studies have provided fairly accurate predictions using only fingerprints. However, the prediction accuracy of our models exceeded the similar previous work in all evaluation metrics. MCC values in our results were 4–41% higher for the different classification tasks. The most challenging task was the discrimination between allosteric and non-allosteric inhibitors, as few samples were available, but our model achieved 19% higher MCC value than the previous study. The less distinction ability between types I and II could be related to the low selectivity among inhibitors of these types. On the other hand, type I$\frac{1}{2}$ inhibitors were highly discriminated with our models as seen in the results when using the I$\frac{1}{2}$ data. The results proved that extending the feature space by using real-valued molecular descriptors, instead of fingerprints, could substantially improve predictions. At the same time, the rational selection of features with dedicated techniques is important to keep reasonable and efficient model performance. It is also expected to help in design and analysis of selective kinase inhibitors. Finally, we could show that machine learning, supported with other computational methods could efficiently help in identifying inhibitor binding modes. The availability of further structural data for kinase-inhibitor complexes is expected to improve predictions.

## Supplementary information

Supplementary captions

Supplementary tables and figures
