# Supplementary material for: Prediction of kinase inhibitors binding modes with machine learning and reduced descriptor sets
Source: Sci Rep. 2021 Jan 12;11:706. doi: 10.1038/s41598-020-80758-4 (PMC7804204; doi:10.1038/s41598-020-80758-4)
Supplement: Supplementary file 1 — Supplementary captions [file 41598_2020_80758_MOESM1_ESM.docx]

**Supplementary Information**

**Title:**

Prediction of Kinase Inhibitors Binding Modes with Machine Learning and Reduced Descriptor Sets

**Authors**

Ibrahim Abdelbaky ^1,2^, Hilal Tayara^3,*^, and Kil To Chong^1,4,*^

^1^Department of Electronics and Information Engineering, Jeonbuk National University, Jeonju 54896, South Korea

^2^Agricultural Research Center, Giza, 12619, Egypt

^3^School of International Engineering and Science, Jeonbuk National University, Jeonju 54896, South Korea

^4^Advanced Electronics and Information Research Center, Jeonbuk National University, Jeonju 54896, South Korea

*Correspondences: Hilal Tayara (hilaltayara@jbnu.ac.kr) and Kil To Chong (kitchong@jbnu.ac.kr)

**Legends for Supplementary Data:**

**(1) Supplementary Figure, and (9) Supplementary Tables are available as sheets in one Excel file: SupplementaryMaterial.xlsx**

**Figure S1:** ROC curves for the results of the classification tasks

**Table S1:** Results of Individual Descriptor Sets (SVC)

**Table S2:** Results of SVC models using 80:20 ratio data splits

**Table S3:** Results of Other ML Techniques (RF: Random Forest, GB: GradientBoosting, LR: Linear Regression)

**Table S4:** Final Predictive Features Selected for Classification Task (I-II)

**Table S5:** Final Predictive Features Selected for Classification Task (I-I½)

**Table S6:** Final Predictive Features Selected for Classification Task (II-I½)

**Table S7:** Final Predictive Features Selected for Classification Task (A-(I+II+I½))

**Table S8:** The Frequently Selected Functional Group Descriptors

**Table S9:** Examples of Good and Poor predicted compounds in each type
